# Spontaneous Pneumomediastinum and Subcutaneous Emphysema Associated With Diabetic Ketoacidosis: A Case Report

**DOI:** 10.7759/cureus.61001

**Published:** 2024-05-24

**Authors:** Anab Usman, John Angelopoulos, Rajeev Kumar

**Affiliations:** 1 Internal Medicine, Bedfordshire Hospitals NHS Foundation Trust, Bedford, GBR; 2 Acute Medicine, Bedfordshire Hospitals NHS Foundation Trust, Bedford, GBR

**Keywords:** macklin phenomenon, hamman syndrome, emphysema, pneumomediastinum, diabetic ketoacidosis (dka)

## Abstract

Hamman's syndrome or Macklin phenomenon - spontaneous pneumomediastinum - is an uncommon condition that often gets missed due to the lack of awareness. It may rarely be associated with diabetic ketoacidosis (DKA) due to repeated vomiting or Kussmaul breathing associated with it. This condition is self-resolving, and improvement in symptoms is usually observed with appropriate management of DKA. Secondary pneumomediastinum is relatively more common, but spontaneous pneumomediastinum, which is rare, is often diagnosed incidentally. Here, we describe a case of a 24-year-old gentleman where this condition was found incidentally during the examination and was confirmed through imaging (X-ray and CT scans) and resolved with successful management of DKA.

## Introduction

Pneumomediastinum is a term based on two words - pneumo means air, and mediastinum means upper chest cavity extending from sternum to the vertebral column. It is described as a condition of the respiratory system when the air enters the chest cavity from the trachea, bronchi, lungs, esophagus and peritoneal cavity and starts accumulating mainly in the neck or abdomen [1]. The aetiology mainly involves blunt or penetrating trauma to the chest wall or oesophagus, tracheobronchial surgery or perforation, infections such as tuberculosis or mediastinitis, interstitial lung disease, and connective tissue disorders [2,3]. Very rarely, it may also occur spontaneously in relation to different diseases and medical conditions [4,5].

In 1939, Hamman described the first comprehensive report on pneumomediastinum. Spontaneous pneumomediastinum, also known as Hamman's or Macklin's syndrome, is a combination of subcutaneous emphysema (air in subcutaneous tissue) and pneumomediastinum. It has been described as a rare complication of DKA and often does not get diagnosed as symptoms such as shortness of breath and chest pain can also be associated with DKA [6,7].

The primary objective of this study was to emphasize the importance of recognizing Hamman's syndrome for early diagnosis of pneumomediastinum, which can help prevent complications in the patient and lead to better disease outcomes. This case report highlighted the incidental discovery of Hamman's syndrome in a patient with symptoms of DKA and discussed the self-resolution of the condition with appropriate medical management. Despite the benign nature and rarity of the condition, the significance of thorough examination in these cases cannot be ignored.

## Case presentation

A 24-year-old male, with a background of type 1 diabetes mellitus (TIDM), presented in the emergency department feeling generally unwell for the previous three to four days with flulike symptoms and temperature. His blood sugar on admission was 20.7 mmol/L with ketones of 5.8 mmol/L suggesting a diagnosis of DKA. He described typical hyperosmolar symptoms but denied any chest pain or palpitations. Additionally, he complained of shortness of breath for one day preceding his admission.

On initial examination, he was alert and oriented but was noted to be dehydrated and tachycardiac. Vital signs showed high temperature, indicative of fever, with high respiratory rate, elevated blood pressure, and increased heart rate. His chest on auscultation was clear, and heart sounds were normal showing no acute respiratory or cardiac compromise, as evident in Table 1.

**Table 1:** Vital monitoring of the patient Comparison of the vital observations at different time intervals.

Parameters observed	At the time of admission	After overnight treatment	Standard range
GCS	15/15	15/15	15/15 (alert)
Blood pressure (mmHg)	160/84	152/100	111-219 systolic
Temperature (°C)	38	36.4	36.1-38.0
Heart rate (beats/min)	145	97	51-90
Respiratory rate (breaths/min)	35	18	12-20
Oxygen saturation (on air)	99%	98%	>96%

The first arterial blood gas (ABG) showed low pH, high glucose, and low HCO_3_( bicarbonate), with increased base excess indicating severe metabolic acidosis as a result of DKA. The elevated ketones of 5.8 mmol/L with raised blood glucose further confirmed diabetic ketoacidosis as a contributing factor. His HbA1C (glycated haemoglobin) was found to be 101 mmol/mol revealing poor diabetic control in background (Table 2).

**Table 2:** Monitoring of arterial blood gas During the treatment of the patient, drastic changes were observed after seven hours of the treatment. *Kilopascal; **partial pressure of carbon dioxide; ***partial pressure of oxygen; ****millimoles per litre

Time into treatment	pH	pCO_2_** (kPa*)	pO_2_*** (kPa)	Glucose (mmol/L****)	Lactate (mmol/L)	Bicarbonate (mmol/L)	Base excess (mmol/L)
Standard range	7.35-7.45	4.5-6.1	12-15	4-10	0.6-2.5	22-29	-2 - (+2)
0 hrs	7.06	1.87	15.3	20.7	1.2	4.0	-24.2
4 hrs	7.00	2.09	14.5	23.9	2.2	3.8	-25.9
7hrs	7.08	2.43	15.9	18.5	3.1	5.4	-22.8
24 hrs	7.34	2.97	10.8	16.5	0.8	12.0	-11.7
48 hrs	7.43	4.90	13.0	13.8	1.5	24.1	0.1

He was managed for DKA as per standard guidelines with intravenous fluids and insulin and showed significant improvement over the ensuing few hours but remained short of breath.

On examination the following day, he was found to have subcutaneous emphysema bilaterally on the upper part of the chest with normal breathing sounds. His vital parameters improved and were noted to be within normal range (Table 1).

**Figure 1:**
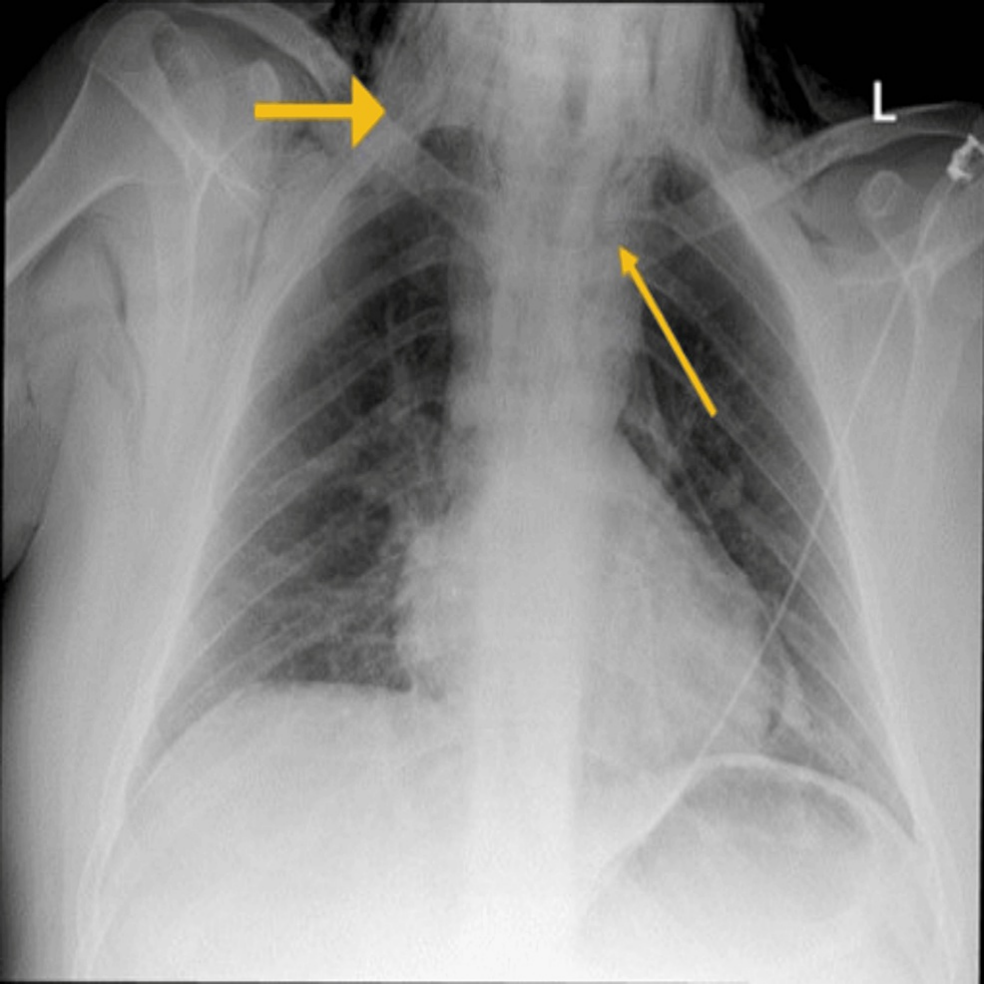
An anterio-posterior view of chest X-ray Arrows showing the location of extensive subcutaneous emphysema extending into the neck with evidence of pneumomediastinum.

**Figure 2:**
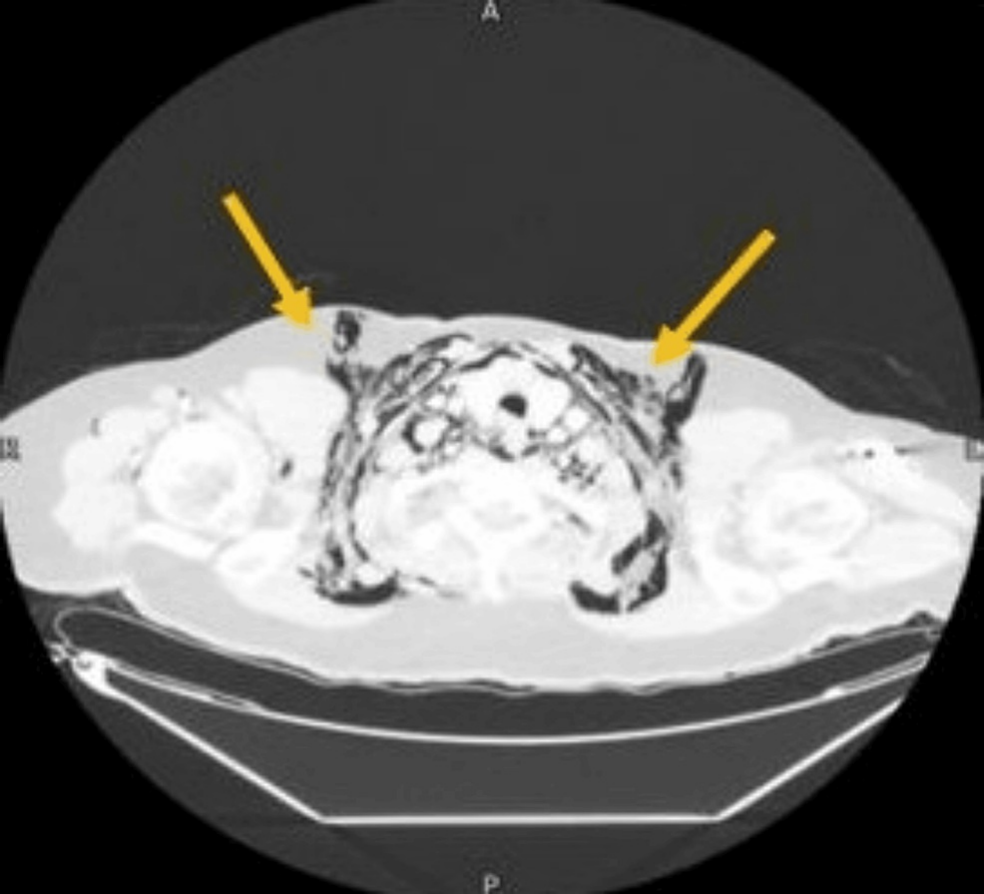
Computed tomography scan of the thorax – coronal view CT scan done on admission showing extensive air in the mediastinum bilaterally, mainly on the right side.

**Figure 3:**
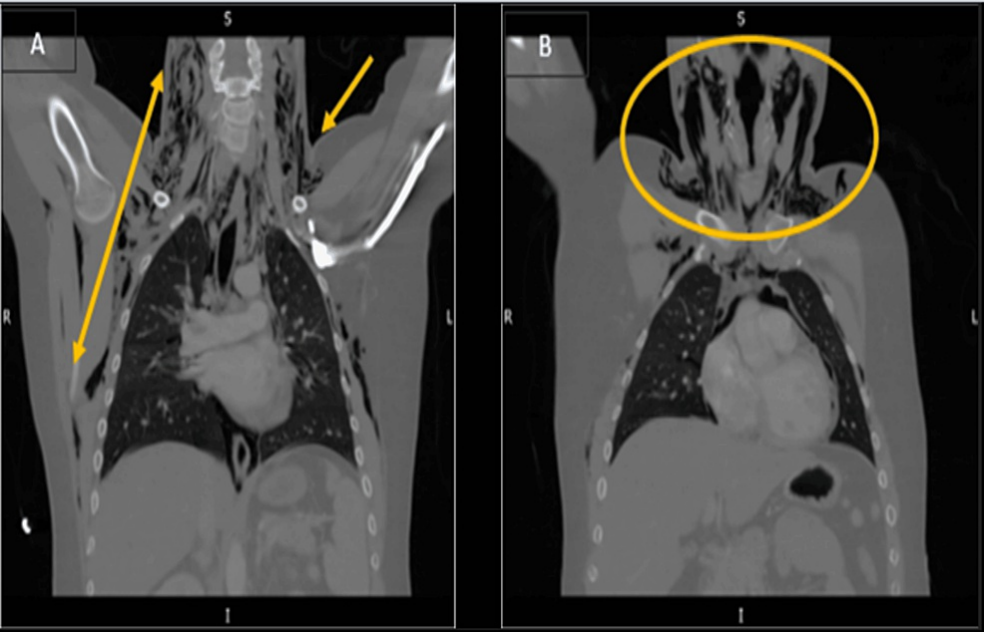
Sagittal views of computed tomography thorax (A) Arrows indicating extensive pneumomediastinum extending to the neck with lung fissures present more on the right side than the left. (B) Area of the neck showing the presence of air in subcutaneous tissue.

A chest X-ray revealed pneumediastinum and subcutaneous emphysema extending into the neck (Figure 1), which was confirmed on a CT scan (Figures 2-3). There was no history of alcohol abuse, and the patient did not have any vomiting or cough to explain the findings. After discussion with the cardiothoracic team, bronchoscopy and gastroscopy were performed, which ruled out perforation.

No other factors were identified that could contribute to the patient’s pneumomediastinum and subcutaneous emphysema.

**Figure 4:**
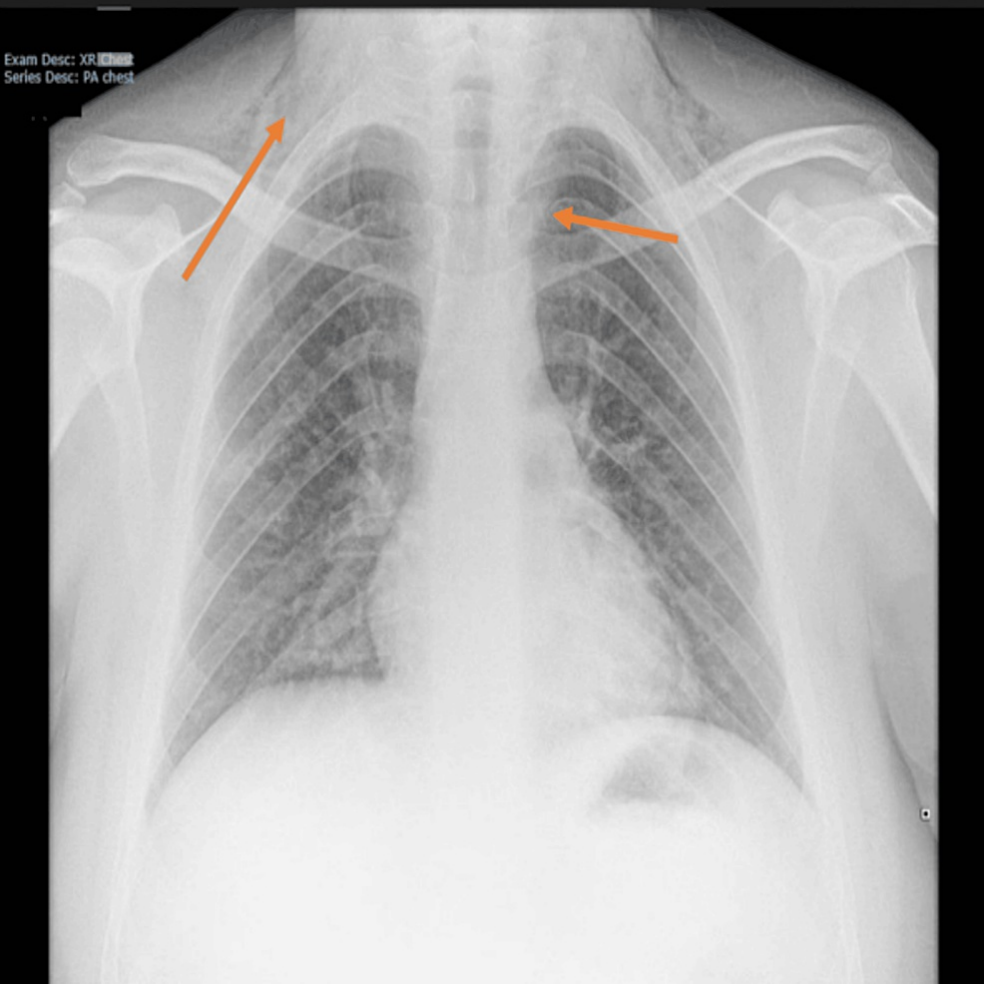
Repeat chest X-ray after one week In comparison with Figure 1, the repeat chest X-ray shows a significant decrease in pneumomediastinum.

The patient was managed conservatively and was observed closely. A repeat chest X-ray a week later showed improvement and resolution of pneumomediastinum (Figure 4). The patient was reviewed by the diabetes specialist nurse daily and was discharged from the hospital after complete recovery with follow-up in the community for his diabetes.

The only obvious causative factor was thought to be hyperventilation, and it was concluded that the patient had spontaneous pneumomediastinum with subcutaneous emphysema due to hyperventilation associated with DKA.

## Discussion

This case report highlights a unique and unexpected complication, Hamman's syndrome, associated with DKA and was an incidental finding on examination [6].

Spontaneous pneumomediastinum associated with DKA is a rare and benign, self-limiting condition. It presents with non-specific symptoms such as tachycardia, shortness of breath, or sometimes chest or neck pain [1]. It is very important to exclude oesophageal rupture, which has a high mortality rate and may sometimes be present in DKA due to recurrent vomiting [4,6].

Spontaneous pneumomediastinum is more common in young adults, most likely due to the fact that mediastinum is made of soft loose tissue, making air penetration easier, which changes to fibrous tissue with age [2]. The study reported by the Cleveland Clinic under the overview of pneumomediastinum illustrates that it is uncommon and involves only one in 25,000 people ranging in age from 5 years to 34 years [8].

The pathophysiology associated with the process is called the Macklin phenomenon, which consists of a rise in intra-alveolar pressure (e.g., due to excessive vomiting, coughing, and hyperventilation), leading to alveolar rupture, air entry into peri-bronchial and perivascular sheaths, and spread of air into the mediastinum and connecting soft tissue [7].

In the majority of cases, this condition is self-resolving with supportive management and correction of DKA and has an excellent prognosis. However, in some instances, it could be related to complications such as Boerhaave’s syndrome, which is related to excessive vomiting or severe chest infection [9,10]. If identified on time and investigated appropriately, these complications can easily be addressed and managed.

Though it has a good prognosis with its self-limiting nature, one might worry about it being missed while treating a patient with DKA, if not identified through thorough examinations and investigations [11].

## Conclusions

Our case report highlights the importance of a detailed and thorough examination of patients presenting with DKA and will hopefully spread awareness among healthcare professionals about this rare complication of DKA leading to early and appropriate diagnosis and improved patient outcomes.

Given the self-limiting nature of spontaneous pneumomediastinum, more focus should be placed on conservative management, and unnecessary investigations should be avoided as they incur additional healthcare costs and also subject the patient to unwarranted risks.
